# A circle of life: platelet and megakaryocyte cytoskeleton dynamics in health and disease

**DOI:** 10.1098/rsob.240041

**Published:** 2024-06-05

**Authors:** Haonan Liu, Julie P. I. Welburn

**Affiliations:** ^1^ Wellcome Trust Centre for Cell Biology, School of Biological Sciences, University of Edinburgh, Edinburgh EH9 3BF, UK

**Keywords:** microtubule, platelet, tubulin, motors, pathogenesis, megakaryocyte

## Abstract

Platelets are blood cells derived from megakaryocytes that play a central role in regulating haemostasis and vascular integrity. The microtubule cytoskeleton of megakaryocytes undergoes a critical dynamic reorganization during cycles of endomitosis and platelet biogenesis. Quiescent platelets have a discoid shape maintained by a marginal band composed of microtubule bundles, which undergoes remarkable remodelling during platelet activation, driving shape change and platelet function. Disrupting or enhancing this process can cause platelet dysfunction such as bleeding disorders or thrombosis. However, little is known about the molecular mechanisms underlying the reorganization of the cytoskeleton in the platelet lineage. Recent studies indicate that the emergence of a unique platelet tubulin code and specific pathogenic tubulin mutations cause platelet defects and bleeding disorders. Frequently, these mutations exhibit dominant negative effects, offering valuable insights into both platelet disease mechanisms and the functioning of tubulins. This review will highlight our current understanding of the role of the microtubule cytoskeleton in the life and death of platelets, along with its relevance to platelet disorders.

## 1. Introduction

Platelets are small, anucleated cells that respond rapidly to blood vessel injury and stop bleeding [1]. Circulating platelets have a short lifespan of 8–10 days before they are degraded in the liver and spleen [2]. The human body releases 1 million platelets per second to maintain an average platelet count of 150–450 million per millilitre of blood [3]. Megakaryocytes differentiated from haematopoietic stem cells are responsible for mass-producing platelets in the bone marrow and the lungs [4]. Circulating platelets are kept resting with a characteristic discoid shape, maintained by a ring of microtubule bundles called the marginal band [5]. Platelets are quickly activated upon damage to blood vessels and undergo a dramatic disc-to-sphere morphological change via reorganizing their cytoskeleton [6]. Recent studies suggest that platelet functions extend beyond haemostasis, as they are also involved in other physiological processes, including inflammation, immune response and wound healing [7–9]. Anti-platelet drugs such as aspirin and clopidogrel can reduce patients’ risk of cardiovascular diseases [10]. However, this may cause acquired platelet disorders, often linked to excessive bleeding from minor injuries or spontaneous bleeding [11]. Under pathological conditions, platelets contribute to tumour metastasis, chronic inflammation, diabetes and Alzheimer’s disease [12–15]. Megakaryocyte maturation and subsequent platelet formation also strongly depend on the dynamic remodelling of the cytoskeleton [16]. In particular, microtubules play a pivotal role in *in vitro* platelet biogenesis. How they contribute and enable platelets to swiftly change morphology under different microenvironments is still unclear. New insights into the molecular mechanisms governing these microtubule-based processes can contribute to developing novel therapeutic strategies targeting platelet-related disorders. This review will discuss the biology of the megakaryocyte and platelet microtubule cytoskeleton in health and diseases.

## 2. Molecular insights into tubulin diversity in platelets

### 2.1. Discovery of tubulin isotypes in platelets

Microtubules are dynamic cytoskeletal assemblies made from α-tubulin and β-tubulin heterodimers that undergo growth and shrinkage, known as dynamic instability [17]. This property is essential for microtubules to build a mitotic spindle and segregate chromosomes, drive intracellular transport, regulate cell architecture and many other aspects of cellular functions in eukaryotic cells [18]. α/β-tubulin heterodimers polymerize into protofilaments that assemble into hollow cylindrical microtubules [19]. Microtubules consist of 13 protofilaments on average, with a diameter of approximately 25 nm and a length of 1–10 μm [20]. Nine genes have been identified for each α- and β-tubulin isotype in humans, and expression of different tubulin isotypes is restricted to tissues and cell types [21]. The first observation of microtubules in megakaryocytes and platelets was made in the 1960s using electron microscopy [22,23]. Microtubule bundles were found to drive proplatelet elongation in megakaryocytes and maintain the discoid shape of platelets [5,24,25]. A quantitative proteomic study of human platelets found multiple α-tubulin isotypes expressed (TUBA1A, TUBA1B, TUBA1C, TUBA3C, TUBA4A and TUBA8) and β-tubulin isotypes expressed (TUBB1, TUBB2A, TUBB2B, TUBB2C, TUBB3, TUBB4, TUBB5, TUBB6 and TUBB8) [26].

The role of α-tubulin isotypes in platelet formation and function was not studied until recently. Strassel *et al*. demonstrated that the levels of α1-tubulin, α4A-tubulin and α8-tubulin increase in cultured megakaryocytes during differentiation stages [27,28]. They found that α4A-tubulin and α8-tubulin account for 29% and 2% of the total α-tubulin in platelet lysate, respectively, by mass spectrometry [28]. Missense mutations in α4A-tubulin are associated with macrothrombocytopenia in mice and patients [28]. More recently, the role of α8-tubulin in platelet formation was highlighted by a high-throughput sequencing study of 448 blood donors with congenital macrothrombocytopaenia who have reduced platelet numbers and enlarged platelets. Six novel mutations in α8-tubulin were identified, and expression of these mutants in U2OS cells disrupts α8-tubulin incorporation into the microtubule to different degrees [27].

β1-Tubulin is the major β-tubulin isotype expressed in megakaryocytes and platelets and is restricted to haematopoietic cells [29]. It is the most divergent β-tubulin isoform, sharing 78% of amino acid similarity with other β-tubulins [29]. The expression of β1-tubulin is tightly controlled by the erythroid transcription factor NF-E2 [30]. NF-E2 gene knockout in mice abolishes β1-tubulin expression and causes macrothrombocytopaenia [31,32]. Platelets from β1-tubulin^−/−^ mice lose their discoid shape, forming an abnormal marginal band prone to break. The overexpression of β2-tubulin or β5-tubulin did not rescue the phenotype, indicating β1-tubulin is not redundant with other β-tubulins [33]. Several mutations in β1-tubulin have been linked to macrothrombocytopaenia, bleeding disorders and thyroid dysgenesis in human patients [30,33,34]. Overexpressing β1-tubulin in Chinese hamster ovary cells showed curved microtubules forming a marginal band-like structure close to the cell cortex [35]. Similarly, overexpression of α8-tubulin increases the microtubule polymerization rate and reduces microtubule straightness in Neuro-2a mouse neuroblast cells [36]. In addition to α- and β-tubulins, an immunofluorescence study demonstrated that murine platelets have γ-tubulin localized at multiple sites on the marginal band. Platelet marginal band microtubules are nucleated despite the lack of a microtubule-organizing centre [37].

### 2.2. Tubulin post-translational modifications in megakaryocytes and platelets

The combination of differential expression of tubulin isotypes and post-translational modifications is referred to as the ‘tubulin code’ [38]. The tubulin code influences microtubule dynamics and interactions with microtubule-associated proteins (MAPs) and motor proteins [39]. Several tubulin post-translational modifications have been identified in the platelet lineage, including phosphorylation, acetylation, glutamylation, tyrosination and glycylation [40–43]. Resting platelet marginal band microtubules are heavily acetylated, in which acetylation has been shown to enhance kinesin-1 and dynein motor recruitment to the microtubule as well as their motility. Platelets in which histone deacetylase 6 is knockout, display microtubule hyperacetylation and have faster platelet activation [44,45]. Polyglutamylation of microtubules adds negative charges to microtubule C-terminal unstructured tails, which facilitates dynein recruitment and movement [46]. The marginal band becomes polyglutamylated during platelet activation, enabling dynein to walk and slide the microtubules apart [47]. However, the role of post-translational modifications in platelet lineage cells remains poorly understood owing to difficulties in genetically manipulating these cells.

### 2.3. Tubulin pathogenic mutations in platelets

Mutations in TUBA4A, TUBA8 and TUBB1 have been linked to macrothrombocytopaenia, a bleeding defect that has enlarged (macro) platelets, low platelet count (thrombocytopenia) and a prolonged bleeding time, which can be inherited or acquired [48–51]. Some tubulin mutations may impair the structure and folding of tubulins, disrupt tubulin heterodimer formation and reduce the functioning tubulin levels in the cell [34,52–54]. However, many pathogenic tubulin mutations are dominant negative [55,56]. Here, we discuss the cellular and molecular mechanisms of cytoskeletal mutations that cause platelet disorders, which may add knowledge for future treatment of bleeding defects.

**Figure 1.**
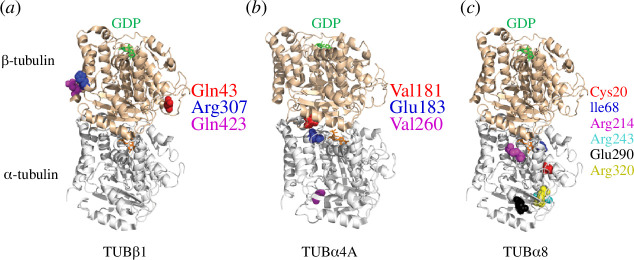
Structural location of the most frequent tubulin mutations associated with platelet disorders mapped onto a tubulin heterodimer. Amino acid residues in which frequent mutations have been identified to cause platelet disorders are highlighted (*a*) β1-tubulin (*b*) α4-tubulin and (*c*) α8-tubulin in different colours (α-tubulin in white and β-tubulin in brown). Orange and green sticks show GTP/GDP nucleotide on α- and β-tubulins, respectively. The three-dimensional structure of tubulin heterodimers was visualized by PyMOL using PDB ID 1JFF.

Electron microscopy studies discovered that the absence of a marginal band and the formation of spherical platelets are associated with a congenital bleeding disorder [57]. Treatment with the microtubule-stabilizing drug Taxol rescues the formation of microtubules in defective platelets. Still, it did not fully restore a marginal band structure, and the cause for the absence of a marginal band was not established [57]. The first pathogenic tubulin mutation was found in the amino-terminal region of β1-tubulin, which we mapped onto tubulin heterodimers (figure 1*a*; PDB: 1JFF). Gln43Pro was identified from a genetic analysis of 33 unrelated patients with congenital macrothrombocytopaenia [58]. Enlarged and spherical platelets were observed by microscopy, decreased levels of β1-tubulin were found by immunoblotting, and impaired platelet adhesion, aggregation and granule secretion were also reported. Transfection of GFP-Gln43Pro-TUBB1 into cultured megakaryocytes resulted in abnormal marginal band formation [58]. The authors suggested that the Gln43Pro mutation in TUBB1 might show some protective effects as this mutation is more prevalent in healthy controls than in men with cardiovascular diseases. Conversely, Navarro-Núñez *et al*. found no cardiovascular protective effect of Gln43Pro, and the mutation is associated with an increased risk of intracerebral haemorrhage in men [59,60]. The TUBB1-Gln43Pro mutation is a common variant with a high minor allele frequency (MAF) of 7.71% in the ExAC genome browser [61].

Several missense mutations in the β1-tubulin structured region were identified in animal and human patient studies [55]. TUBB1-Asp249Asn was identified as a frequent mutation in Cavalier King Charles spaniels associated with macrothrombocytopaenia [62,63]. Using whole-genome sequencing and an absolute immature platelet fraction assay that measures platelet production, a TUBB1-Arg307His mutation was identified in patients with immune thrombocytopenia that showed increased platelet destruction and reduced platelet production [64]. A subsequent investigation conducted by the same research group revealed that the TUBB-Arg307His mutation caused a significant decline in platelet number and a decreased rate of microtubule regrowth after nocodazole-induced microtubule depolymerization, suggesting this mutation impairs microtubule stability [65]. The TUBB1-Arg307His mutation is another common mutation with a high MAF of 14.7% [61]. This mutation is located in the intermediate region of the tubulin-binding domain (figure 1*a*; electronic supplementary material, figure S1*a*). Intriguingly, multiple sequence alignments between *Homo sapiens* tubulin isotypes show that all the other β-tubulin isoforms at residue 307 have a histidine instead of arginine except β1-tubulin [55] (electronic supplementary material, figure S1*b*). Moreover, β1-tubulin sequence alignment across 13 metazoan species shows that this arginine at residue 307 is highly conserved (electronic supplementary material, figure S1*c*). It is currently unknown whether the residue’s identity at position 307 can cause functional differences in different cell types.

A β1-tubulin C-terminal mutation Glu423* was found close to the flexible tail that replaces glutamine with an early stop codon and truncates the protein, resulting in thrombocytopenia (figure 1*a*) [66]. Immunostaining showed an enlarged marginal band and reduced tubulin signal from the patient’s platelets. The authors hypothesize that the mutation might affect protein folding by disrupting the H12 helix, preventing heterodimer assembly and reducing tubulin intracellular levels [67].

In α-tubulin, a missense mutation TUBA4A-Val260Glu was identified in mice from a mutagenesis screen, which causes a 21% decrease in platelet count and a 149% increase in mean platelet volume [28]. Subsequent screening from blood donors with a low platelet count identified an individual carrying both Val181Met and Glu183Gln mutations in TUBA4A. As shown in figure 1*b*, Val260 and Val181 are located at the α- and β-tubulin interdimer interface, so these two mutations might disrupt the tubulin heterodimer formation and impair microtubule assembly. The patient’s platelets have loosely assembled marginal bands and lack the discoid shape, although the platelet count is average. A follow-up study demonstrated that the size or number of platelets was unaffected in α4A-tubulin knockout mice. However, knockout of both α4A- and β1-tubulins in mice significantly reduced the number of microtubules present in the platelet and caused the platelet to become spherical [68]. Megakaryocyte maturation, proplatelet extension formation, and *in vitro* platelet adhesion were severely impaired in tubulin double-knockout mice [68]. More recently, six novel TUBA8 mutations—Cys20Tyr, Ile68Leu, Arg214Cys, Arg243Cys, Glu290Lys and Arg320Trp—were identified in patients with congenital macrothrombocytopaenia [27]. The marginal band microtubules of these patients’ platelets were scattered at the cell periphery, which caused platelets to adopt a round shape. Transfection of these α8-tubulin mutant isotypes into U2OS cells showed reduced or no incorporation into microtubules. We mapped these mutations onto a tubulin heterodimer structure (figure 1*c*; PDB: 1JFF). Cys20tyr Ile68Leu mutations occur in the hydrophobic core of the α-tubulin subunit and may impact the structure and stability of tubulin-α8. Mutations Arg214Cys, Arg243Cys, Glu290Lys and Arg320Trp are found on the surface of tubulin-α8. These mutations may not affect tubulin heterodimer structure or microtubule assembly, but instead interfere with how the microtubule interacts with associated proteins.

In summary, a growing number of pathogenic tubulin mutations that affect platelet function and physiology have been identified, facilitated by the increased access to whole-genome sequencing. While some mutations affect the stability of tubulin heterodimers and microtubules, some mutations appear dominant negative and the pathogenic mechanism is currently unknown. Generally, the mutations interfere with megakaryocyte maturation, disrupt platelet production, impair platelet activation and induce structural abnormalities in platelets, contributing to bleeding disorders. Understanding the mechanistic cell biology of platelets and megakaryocytes is essential to manage and treat these disorders with precision medicine.

## 3. Role of microtubules in megakaryocyte and platelet formation

Microtubules play a pivotal role in the differentiation and proliferation process of haematopoietic cells. They support the maturation of megakaryocytes from haematopoietic stem cells and provide forces for megakaryocyte cytoplasm remodelling, maintaining resting discoid platelet shape and triggering the shape change during platelet activation. Growing evidence suggests that microtubule-dependent platelet formation and function are essential for maintaining normal haemostasis.

### 3.1. Microtubules assemble into a multipolar spindle during megakaryocyte endomitosis

Megakaryocytes are polyploid cells residing in the bone marrow. Cells that reach polyploidy through consecutive rounds of DNA replication without mitosis go through endoreplication [69,70]. In megakaryocytes, the chromosomes condense and segregate (typically endomitosis) but without cytokinesis [71–73]. A mature megakaryocyte contains up to 128N DNA with an enlarged cell size of 100 μm. Intriguingly, while megakaryocytes can undergo up to six rounds of endomitosis, most megakaryocytes in the bone marrow repeat only three rounds of endomitosis to reach 16N DNA [74]. Polyploidization allows megakaryocytes to undergo functional gene amplification and increase intracellular ribosome concentration to enhance protein synthesis, which is essential to meet the secretory functions of platelets [74–76]. Polyploid megakaryocytes contain multilocular nuclei and multiple centrosomes, and their cytoplasm comprises platelet-specific organelles and cytoskeletal proteins [73,77,78].

**Figure 2.**
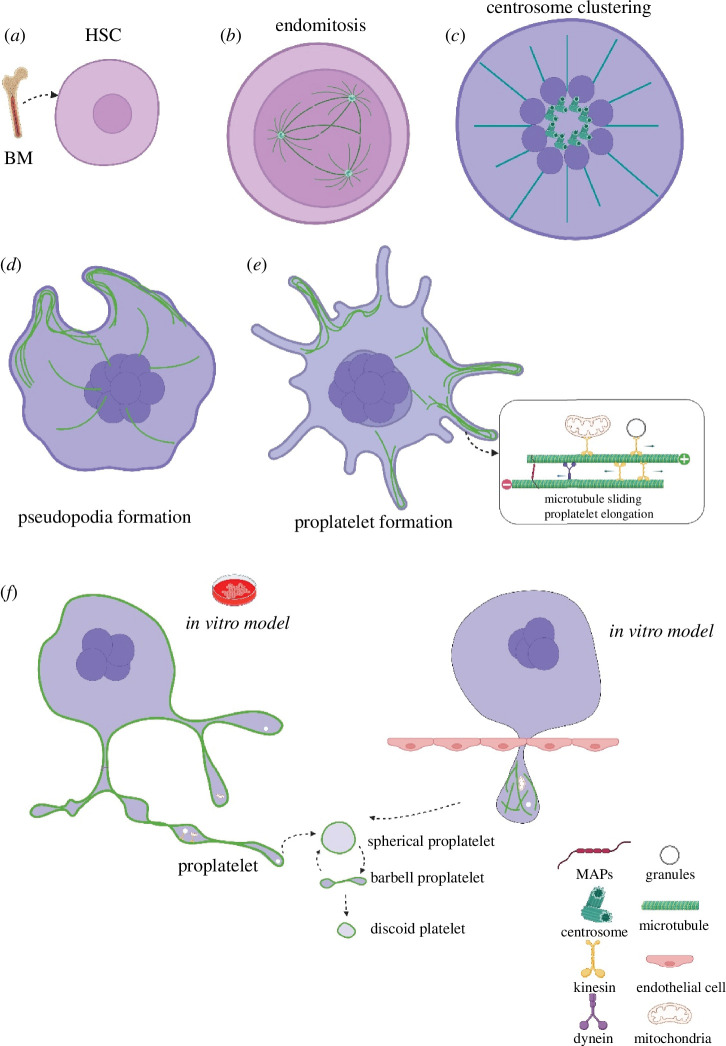
Schematic of microtubule function during *in vitro* and *in vivo* platelet formation from megakaryocytes. (***a***) Megakaryocytes differentiate from haematopoietic stem cells within the bone marrow, which is regulated by thrombopoietin (TPO). (*b*) Megakaryocytes undergo endomitosis and maturation, resulting in polyploidy, enlarged cell size and platelet-specific protein synthesis. Microtubules are assembled into multiple spindles that support asymmetric chromosome segregation. (*c*) The sliding of spindle microtubules drives centrosome clustering within a typical 16N DNA mature megakaryocyte. A monospindle emanates from the microtubule-organizing centre to the cell cortex. (*d*) Before proplatelet formation, megakaryocyte centrosomes cluster into a microtubule-organizing centre, and microtubules relocate to the cell cortex. (*e*) Microtubule bundles are cross-linked by microtubule-associated proteins formed beneath the pseudopodia. Sliding of overlapping microtubules by dynein results in proplatelet elongation. Kinesins transport mitochondria, granules and other organelles into proplatelet far ends and are trapped with the microtubule coil. (*f*) Models for platelet release from megakaryocytes. In the *in vitro* model, megakaryocytes migrate to vascular sinusoids; elongated proplatelets are released into the bloodstream and fragmented into large discoid preplatelets. Preplatelets interconvert into barbell-shaped proplatelets under shear stress—dynamic microtubule reorganization by twisting, merging and folding causes barbell-shaped proplatelets to split into two platelets. In the *in vivo* model, megakaryocytes release large protrusions into sinusoid blood, then fragment into proplatelets or preplatelets in the microcirculation. Figure created using BioRender.

Mitotic spindle poles of endomitotic megakaryocytes are usually multipolar owing to supernumerary centrioles (figure 2) [79]. The mitotic spindle self-assembles and mitotic regulatory proteins, such as the error correction kinase Aurora B and the microtubule anti-parallel cross-linker protein regulating cytokinesis 1, were expressed in cultured megakaryocytes, which subsequently were found to enter telophase [80]. A study examined the dynamics of chromosome segregation in megakaryocytes. Asymmetric chromosome segregation was mediated by multipolar spindles, indicating that anaphase A occurred. However, the spindle poles did not move apart, suggesting that megakaryocytes did not enter anaphase B. Early studies demonstrated that endomitosis was arrested during anaphase B, causing megakaryocytes to skip telophase and cytokinesis [78,79].

Endomitosis seems to be the result of the absence of actinomyosin ring assembly and contraction. RhoA is a small GTPase that activates contractile actin ring assembly [81]. In megakaryocytes, RhoA is not recruited to the cleavage furrow, so the actin ring fails to assemble and cytokinesis does not take place [80,82]. A lack of myosin II accumulation in the midzone causes dysfunction of the contractile ring, which results in failed cytokinesis and furrow regression [80]. Thus, endomitosis in megakaryocytes takes place. Recent work showed that megakaryocyte polyploidization was independent of RhoA [83]. However, RhoA is important for other aspects of megakaryocyte biology, suggesting an active mechanism prevents RhoA recruitment to the furrow to facilitate endomitosis.

Most megakaryocytes in the bone marrow repeat only three rounds of endomitosis to reach 16N DNA, and the mechanism controlling the number of endomitotic cycles is unknown [74]. Moreover, the knockout of the mitotic kinase Plk1 leads to abnormal spindle formation, defective polyploidization and megakaryocyte cell death [84]. This observation aligns with the role of Plk1 in diploid cells, where it regulates spindle bipolarity and cytokinesis by recruiting the RhoGEF Ect2 localization to the central spindle [85]. It is unclear how microtubules assemble a physiological multipolar spindle, establish tension at kinetochores and progress through mitosis without triggering any spindle checkpoint. The strength of the checkpoint may decrease as cell size increases, which would be similar to what has been observed in embryonic stem cells [86]. Some degree of chromosome separation may be sufficient for chromosomes to spread and initiate the next round of DNA replication. Future work could address these questions.

### 3.2. Microtubule motors organize microtubules during platelet formation

The essential role of microtubule in megakaryocyte maturation has been demonstrated by treating megakaryocytes with microtubule-depolymerizing drugs, including nocodazole, colchicine and vincristine, which impaired megakaryocytes maturation and the formation of proplatelets [24,78,87,88].

A challenging task for megakaryocytes is to coordinate a large number of duplicated centrioles during each endoreplication cycle. The microtubule cytoskeleton undergoes dramatic reorganization after megakaryocytes become mature. Once they have reached the last process of endoreplication, centrosomes within megakaryocytes cluster into the cell centre and assemble into a microtubule-organizing centre (figure 2*c*) [89,90]. Megakaryocyte centrosome clustering might be driven by the minus end-directed kinesin-14 motor protein KIFC1. Increased levels of KIFC1 were observed in ovarian cancer cells to promote cell survivability [91,92]. KIFC1 cross-links and slides parallel microtubules, leading to centrosome clustering. NuMA and the minus end-directed motor dynein mediate microtubule minus-end clustering in oocytes lacking centrosomes. The spindle stability also depends on the motor activity of KIFC1 [93]. Before proplatelet formation, the clustered centrosomes disassemble, and microtubules move to line the cell cortex by an unknown mechanism [94]. Megakaryocytes form multiple cytoplasmic extensions of pseudopodia, in which microtubules on the cell cortex begin to cross-link into bundles and elongate underneath the plasma membrane of newly formed pseudopodia (figure 2*d*) [33,94]. These microtubule bundles drive the formation of long, bulky proplatelets with a bulbous head starting at the far end, where microtubules are looped into a coil to form a platelet-shaped structure [95]. This process is also driven by actin polymerization, as treating megakaryocytes with the actin inhibitor cytochalasin abolishes branching and bending of proplatelets [94]. Moreover, the elongation of proplatelets is not to be driven entirely by microtubule polymerization, as proplatelets continue to elongate in the absence of microtubules after nocodazole treatment [96]. Proplatelets from triton-X-100 permeabilized megakaryocytes can still elongate at a rate of 0.65 μm min^−1^ after adding 1 mM adenosine triphosphate (ATP), suggesting microtubule sliding also drives proplatelet growth (figure 2*e*) [96]. Immunofluorescence showed dynein localizing along the proplatelet microtubules, while kinesin was localized to microtubules in the megakaryocyte cell periphery. Overexpression of dynamitin/p50, a subunit of dynactin, leads to dissociation of the p150 subunit from the dynactin complex and disrupts the dynactin–dynein function. Overexpression of dynamitin/p50 inhibits proplatelet elongation from megakaryocytes [96–98]. These studies indicate that the dynein/dynactin complex slides microtubules during proplatelet elongation, and microtubule bundling and sliding are essential for *in vitro* proplatelet formation. However, which MAPs beyond microtubule motors facilitate the bundling and cross-linking of microtubules in proplatelets is not known.

### 3.3. Current model for platelet formation

The proplatelet theory of platelet biogenesis suggests matured megakaryocytes extend long beaded cytoplasmic extensions called proplatelets, which contain several teardrop-shaped, platelet-sized swellings joined by cytoskeletons [99]. Proplatelet structure has been observed both *in vitro* and *in vivo* [24,100].

The microtubule cytoskeleton is crucial in forming proplatelets, the precursors to platelets. Mechanical force is also thought to play a role in releasing platelets from megakaryocytes into the bloodstream by the fragmentation of proplatelets. Proplatelet protrusions penetrate the sinusoidal endothelium into the bloodstream, where they undergo fragmentation owing to a high shear force from the blood flow that promotes the fission of proplatelets and releases discoid platelets directly [99,101,102]. By monitoring cultured megakaryocytes on a microfluidic bioreactor, a physical simulation revealed that actomyosin contractility and microtubule sliding generated elastohydrodynamic instability (similar to the Plateau–Rayleigh instability that explains why the falling stream of liquid fragmented into smaller droplets with the same volume) over proplatelets' bodies, resulting in their fragmentation [103]. However, researchers failed to replicate an efficient platelet formation of 1000 to 3000 platelets per megakaryocyte using *in vitro* cultured megakaryocytes. This suggests we do not fully understand how proplatelets are fragmented into platelets. There may be an alternative mechanism to control *in vivo* platelet release [102,104].

In living mouse bone marrow, a homogeneous distribution of short microtubules was observed from *in vivo* megakaryocyte protrusions, contrasting with long microtubule bundles observed in proplatelets formed from cultured megakaryocytes [95,105]. An *in vivo* intravital imaging study using TUBB1-deficient mice observed that microtubules are not bundled like *in vitro* proplatelet extensions but aligned as an array [106]. However, this is likely to be owing to the reduced tubulin levels present in megakaryocytes. An increased number of megakaryocytes in the bone marrow of TUBB1-deficient mice suggests that a compensatory mechanism is activated. Models for *in vitro* and *in vivo* platelet formation remain to be reconciled. Further studies on the molecular mechanisms that control platelet biogenesis will improve our knowledge towards the *ex vivo* generation of platelets.

## 4. Microtubules regulate the shape change of platelets

The discoid shape and small cell size of platelets enable them to pass flexibly through narrow capillaries and flow along the blood vessel wall to monitor vascular integrity [107,108]. An injury in the blood vessel will cause platelet activation within seconds. Activated platelets swiftly remodel their cytoskeleton to change from discoid into a spherical shape, form cellular protrusions, adhere to injured surfaces and spread out onto injured sites [109]. This remodelling is vital for the acute haemostasis response.

### 4.1. Microtubules maintain the discoid shape of resting platelets

Platelets are small (0.5 × 3 µm) anucleated cell fragments that do not have a nucleus or a microtubule-organizing centre [110]. Microtubules were first observed in the 1960s by electron microscopy in the platelets of hamsters, rats and humans [23,111,112]. Five to 20 microtubules were seen in cross sections of platelets in electron micrographs in the marginal band close to the plasma membrane. Cold treatment confirmed they were microtubules [112]. These studies suggested that the microtubules may play a structural role, but it was unclear whether the marginal band was made from one single long microtubule or multiple cross-linked. Studies measuring dynamic microtubule plus ends in platelets evaluated the number of individual microtubules per marginal band to 6–10 microtubules on average [37]. The disc-shaped platelet is only found in mammalians. In contrast, nucleated thrombocytes in nonmammalian species, such as zebrafish, are spindle- or oval-shaped [113,114]. Several studies proposed that this marginal band is essential for maintaining the resting platelets’ shape, size and function. Marginal band disassembly using cold treatment to depolymerize microtubules caused a reversible change from discoid to spherical shape [112,115,116]. Initially, researchers believed the marginal band was formed by a long, single continuous microtubule of 100 μm in length coiling several times [117–120]. However, live-cell imaging of platelets using GFP-tagged end-binding proteins EB1/EB3 demonstrated that platelets contain dynamic microtubules nucleated by γ-tubulin with mixed polarity [37]. The average microtubule growth rate in platelets was 7.7 μm min^−1^ with a range measured between 2.4 and 12.2 μm min^−1^. In most cells, microtubules have an average growth rate of 12–24 μm min^−1^ (reviewed in Ref. [121]). Using EB3-GFP, it was reported that microtubules grow at 13.2 μm min^−1^ in neuronal cells, but the speed doubles to 26.4 μm min^−1^ in glia and cultured COS1 cells [122]. In HeLa cells, microtubules were shown to grow at an average speed of 7 μm min^−1^ [122]. As the speed of EB3-GFP comet reflects the average microtubule growth rate, all growing microtubules should have a similar elongation rate; thus, the variation in platelet microtubule growth speed implies microtubule sliding might occur in platelets [37]. Platelets contain acetylated and polyglutamylated tubulin, indicating that dynamic and stable microtubules are both present in the marginal band [37]. Together, these findings suggest that the marginal band is a stable, self-assembled structure that is also highly dynamic, similar to the mitotic spindle.

Platelets are mechanically resilient and withstand the shear forces of blood flow. Dmitrieff *et al*. recently established a mathematical model to examine how mechanical forces balance marginal band rigidity and cortical tension in erythrocytes and platelets using data collected from 25 species [123,124]. They predicted that erythrocyte size is correlated with microtubule length and rigidity but is inversely correlated with cortical tension. In their model, marginal band coiling during platelet activation is owing to cortical tension increasing faster than microtubule cross-linkers can unbind [123]. In a separate study, live imaging of platelets in a microfluidic system in combination with electron tomography and super-resolution microscopy revealed that the amount of polymerized tubulin determines the platelet size, and they estimated the size of microtubule cross-linkers in the marginal band to be around 5–6.25 nm [125]. Whether microtubules are cross-linked in the platelet to provide stability for the marginal band is still unclear. KIFC1, present in megakaryocytes and platelets, is a good candidate to stabilize the marginal band of platelets. KIFC1 is known to cross-link and slide both parallel and anti-parallel microtubules using its motor domain and C-terminal non-motor region [126–128]. In mitotic cells, KIFC1 contributes to spindle formation and stability [126,129]. In neurons, it is important for nuclear migration and neuronal migration [130]. Dynein cross-links and slides anti-parallel microtubules [131], and thus may also play a role in stabilizing the marginal band. Actin filaments, free and bound to cofilin, are also observed in the lumen of microtubules in platelets [132]. It will be important to define the role of lumenal actin on the mechanical properties of platelet microtubules.

Interestingly, a study found that applying mechanical stretch and compression cycles to RPE-1 cells results in curved, stable microtubules forming along the cell periphery, and enucleated RPE-1 cells also have microtubules organized into a circular organization [133]. Their finding suggests that this mechanoresponsive stabilization of microtubules depends on cytoplasmic linker-associated protein 2 (CLASP2). CLASPs have also been reported to bind to curved microtubules at the cell cortex, stabilize microtubules and repair damaged microtubule lattices [134,135]. CLASP2 is essential for haematopoietic stem cell development, and knockout of CLASP2 causes thrombocytopenia in mice [136]. However, the role of CLASPs and many other MAPs in the platelet lineage is still unclear. These studies imply that mechanical forces applied to the cell drive the internal cytoskeletal organization to assemble the marginal band.

### 4.2. Microtubules undergo dynamic reorganization in activated platelets

**Figure 3.**
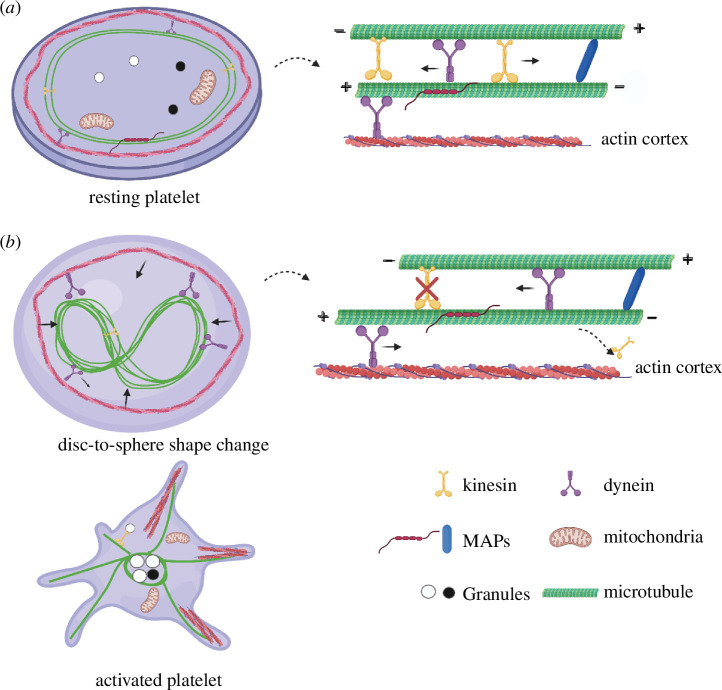
Schematic of marginal band microtubule reorganization during platelet activation. (*a*) Discoid platelets contain a circumferential marginal band of multiple dynamic short microtubules and a stable long microtubule, kept at equilibrium owing to kinesin antagonizing dynein motor activity microtubule-associated proteins cross-linking microtubules. (*b*) Activation of platelets causes inhibition of kinesin activity, in which dynein starts to slide microtubules, resulting in elongation and coiling of the marginal band, leading to compression of the actin cortex and the disc-to-sphere shape change. Polymerization of microtubule and actin filaments results in the formation of filopodia-like cell protrusions that facilitate platelet adhesion and aggregation. Figure created using BioRender.

During activation, platelets change from discoid to spherical and secrete granule contents to activate and aggregate surrounding platelets. They also release cytokines and inflammatory factors to trigger endothelial cell response [137]. This transition requires a rapid reorganization of the cytoskeleton. Using electron and confocal microscopy, studies found that actomyosin contraction and coiling of the marginal band lead to platelet rounding during activation [138–140]. Observation from live-cell imaging of platelets exposed to motor inhibitors demonstrated that marginal band microtubules were maintained at an equilibrium state within resting platelets as the result of a balance between kinesin and dynein. Specifically, inhibition of dynein prevented platelet spreading, whereas inhibition of conventional kinesins with a kinesin ATPase inhibitor aurintricarboxylic acid induces marginal band coiling and causes platelet rounding [141–143]. After platelet activation, the authors proposed that dynein slides microtubules against the actin cortex. This causes elongation of the marginal band, leading to actomyosin contraction and marginal band coiling within the confined cytoplasm space of platelets (figure 3*b*) [143]. The identity of the kinesins that counterbalance dynein is unclear [26,144,145]. KIF5B, KIF13A and KIF13B are plus end-directed kinesin motors in platelets and potential candidates for coordinating the marginal band [146,147].

Emerging research suggests microtubules may play a role in mechanosensing and mechanotransduction by responding to mechanical forces [133,148]. External compressive forces applied to cultured RPE1 cells and enucleated cytoplasts cause microtubule stabilization and the formation of curved microtubules at the cell periphery [133]. This organization is reminiscent of the microtubule organization into the marginal band of platelets. The authors found that the force-generated microtubule-stabilizing effect was dependent on CLASP2 relocation to microtubule shafts [133]. It will be interesting to see if CLASP2 and microtubule plus end-binding proteins are also relocalized along the microtubules in platelets.

Dynein and kinesin activity also control the architecture of microtubule networks, with implications for cell architecture and organization. The balance of forces between kinesin and dynein motor is essential for maintaining the mechanical integrity of the cytoskeleton and regulating cell shape [149–152]. Inhibition of dynein and myosin in MDA-MB-231 cancer cells resulted in the formation of a giant microtubule ring similar to platelet marginal band structure, deformation of the cell into a discoid shape, and the microtubule-organizing centre moving away from the cell centre to the ring [152]. These structural similarities led the authors to propose that the formation of circular microtubule bundles is driven by the disbalance of dynein and kinesin motor activities. When the cells were treated with kinesore (a small-molecule activator that causes kinesin-1 overactivation), an expanding microtubule ring was also observed. They hypothesized that dynein recruits microtubules to cell adhesion sites and drives inward microtubule compaction. At the same time, kinesins counterbalanced dynein by bundling and expanding the microtubule into a marginal band structure [152]. This is in agreement with a study showing antagonistic balance between dynein and kinesin keeps platelets in the resting state. A mathematical model was built based on how over-curved slinky spring rings fold into different saddle shapes using minimum energy, which resembles how platelet marginal band shape changes [153]. Previous studies have also established that the microtubule and actin cytoskeleton are cross-linked in many cell types, including platelets [154–160].

Platelet reorganization is also initiated through changes in small-molecule concentration. Activated platelets release Ca^2+^ with cytosolic-free Ca^2+^ increasing from 0.1 to 1.0 μM [161,162]. Calcium levels may also influence the marginal band in multiple ways. First, calcium causes microtubules to depolymerize [163]. As a result, marginal band rigidity may decrease. Elevated cytosolic Ca^2+^ activates calmodulin, a calcium-binding messenger protein, which activates and induces conformational change in downstream proteins, such as calmodulin-dependent protein kinase [164]. Calmodulin-dependent protein kinase then regulates the activity of kinesin-1 through inhibitory phosphorylation of kinesin light chain or kinesin heavy chain [165–167]. In contrast, increased Ca^2+^ has been shown to enhance cytoplasmic dynein activity and increase microtubule sliding in axonemes isolated from Chlamydomonas [168]. Increased Ca^2+^ causes partial microtubule depolymerization and activates actomyosin filaments to compress the microtubule ring [163].

Disease states such as cancer cause the structural adaptation of platelets. In cancer patients, an increased number of platelets are produced (thrombocytosis), and the platelets often become hyperreactive in that they are activated at a lower threshold [169,170]. A recent study using cryo-electron tomography showed the architecture of platelets in ovarian cancer patients with malignant stage of the disease was altered [171]. The number of mitochondria was increased while the length of microtubules was decreased, causing a disruption of the marginal band. This may destabilize the resting state of platelets. Structural organization of platelets could, therefore, be used as a biomarker in the detection of cancer and other platelet-related diseases.

### 4.3. Packaging and release of granules in platelets

Platelets contain granules, specialized secretory organelles that are released upon activation and secrete hundreds of substances into the blood circulation [172]. The central granules in platelets are α-granules, which contain cytokines and growth factors that facilitate thrombus growth and contribute to inflammatory responses [173]. Defects in α-granule formation during megakaryocyte maturation cause grey platelet syndrome, resulting in mild to moderate bleeding problems [174]. Platelets have dense granules containing ADP, ATP, calcium and serotonin, contributing to coagulation [175]. Dense granule deficiency is associated with bleeding disorders such as Hermansky–Pudlak syndrome and Chediak–Higashi syndrome, causing prolonged bleeding, hypopigmentation and immune defects [176]. Other platelet organelles, such as lysosomes, mitochondria and glycogen, are packaged in megakaryocytes and transported into proplatelets.

In eukaryotic cells, intracellular cargos are transported and distributed by motor proteins, mainly myosins, kinesin and dynein, along cytoskeletal tracks (reviewed by [177]). Fluorescence microscopy imaging of mitochondria and granules in megakaryocytes found organelle movement in proplatelets was bidirectional, with an average speed of 0.2 μm min^−1^ along the microtubule [178,179]. Kinesin-1 has been identified as the plus end-directed motor transporting granules in platelets [180]. Kinesin-coated carboxylated latex beads move bidirectionally in permeabilized proplatelets, suggesting that the trafficking of organelles from megakaryocytes to proplatelets is driven by kinesin activity [179]. However, the speed of organelle movement (0.2 μm min^−1^) is much slower than the *in vitro* kinesin-1 movement speed (around 0.7–0.9 μm s^−1^) reported in the literature [181,182]. The speed discrepancy could be owing to cross-linking of microtubules, cargos bound by motors of opposite directionality or modification of microtubule tracks [183]. Current findings and platelet proteome data suggest that the kinesin-3 family plus end-directed motor KIF13A/B could also be involved in transporting proplatelet organelle [26,184,185]. A recent bioinformatical study integrated six previously published platelet proteome datasets and two genome-wide human transcriptomes, allowing us to predict the theoretical platelet proteome and compare the difference with megakaryocytes [186]. There might be a mechanism in megakaryocytes to deliver specific proteins into proplatelets selectively. Furthermore, assembling a microtubule array after platelet activation may also contribute to the transportation and release of platelet granules [180].

## 5. Concluding remarks

The fundamental cell biology principles underlying the role of microtubules in megakaryocyte maturation, platelet biogenesis and shape change have been investigated for over three decades. Despite these studies, our knowledge of the precise molecular mechanisms governing these processes remains incomplete, largely owing to the challenges posed by some of the unique characteristics of platelets such as a lack of nucleus, a short lifespan and the difficulty of generating genetically modified platelets. One key area of interest is the role of microtubules in the transport of granules and proteins during proplatelet formation and platelet activation. It is essential to elucidate the cellular factors that initiate microtubule relocation and bundling at the tips of proplatelets. Equally important is comprehending the cytoskeletal mechanisms driving marginal band activation through microtubule sliding, twisting and curving during platelet formation, and how pathogenic mutations interfere with these processes.

Addressing these questions will require comprehensive molecular, cellular and biochemical investigations employing advanced techniques to study and manipulate platelets. Such efforts will significantly enhance our knowledge of cellular mechanisms, bridge the gap between *in vitro* and *in vivo* platelet formation and facilitate the future *ex vivo* generation of platelet products. Moreover, these investigations will contribute to the development of anti-platelet drugs for the treatment of platelet-related disorders.

## Data Availability

Electronic supplementary material is available online [187].
